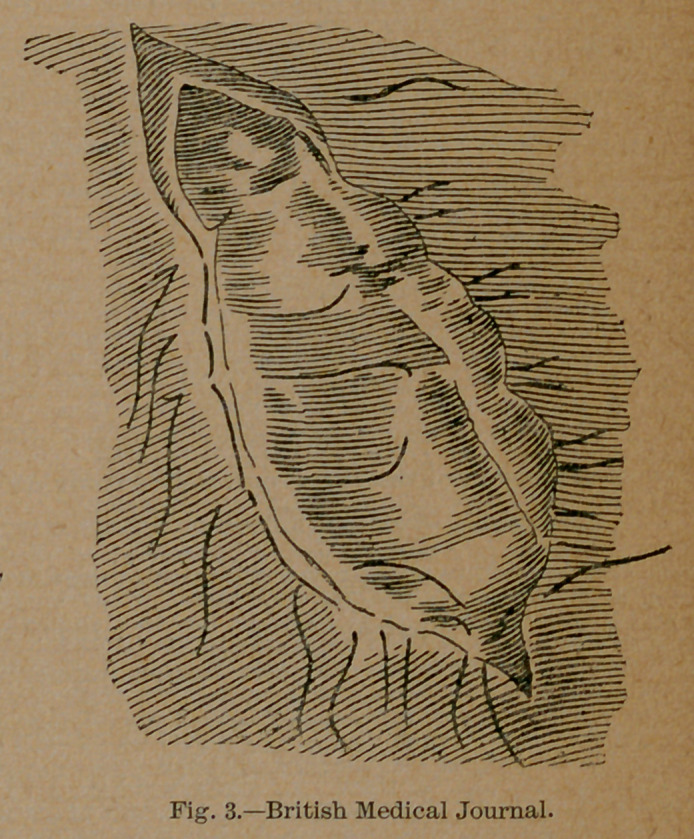# Inguinal Colotomy, with Report of a Case

**Published:** 1891-01

**Authors:** Arch. Dixon

**Affiliations:** Henderson, Ky.


### INGUINAL COLOTOMY, WITH REPORT OF A CASE.

BY ARCH. DIXON, M. D., HENDERSON, KY.

On the 19th day of June, of the present year, I was requested by Dr. P. H. Griffin to examine a case of suspected fecal impaction, with consequent obstruction. The patient, Mr. H——, age 22, tall and spare, had a history of frequent and long-continued attacks of constipation, which had previously yielded to treatment within a short time. On the 12th of June Dr. Griffin had been called to see him in one of these attacks, and had found what he supposed to be a fecal impaction at the sigmoid flexure. There was intense pain in the bowels radiating over the abdomen, and accentuated in the left inguinal region. The "usual remedies" were ordered, but without relief. On the 13th, in addition to calomel, followed by castor oil, large enemata of hot water were used with the effect of producing increased pain and only a small discharge of fecal matter from the rectum. Opiates were given and purgatives administered each day with no relief; he having taken on the 18th, the day previous to my visit, about a pint of castor oil and fully as much Epsom salts. On the morning of the 19th I found him with a pulse of 140, temperature 102, respiration 36; tympanitis and general tenderness over the abdomen. Under a careful examination no tumor could be made out, but on passing the finger into the rectum, a bulging of the bowel, with a teat-like protuberance extending below the sigmoid flexure, could be felt. The gut was entirely occluded. Dr. Griffin's attention was called to this fact, and colotomy proposed as the only relief from the intense pain the patient was suffering. The length of time which had elapsed since the obstruction began, nearly eight days, almost precluded the possibility of recovery, yet I deemed the operation a justifiable one for the relief of the pain, even if nothing more was accomplished. This opinion Dr. Griffin concurred in. The case was laid before the family and the operation accepted. Two hours later, assisted by Drs.

John Young Brown, P. H. Griffin, Wm. A. Quinn and Medical Student Arch. Dixon, Jr., the operation was done. The usual incision was made, followed by a bulging of the small intestine through the opening. This was pushed back and retained by a flat sponge. Two fingers of the right hand were introduced, the rectum sought and followed up to the sigmoid flexure; at this point a distinct tumor could be felt, blocking up the entire gut. Further examination by breaking of adhesions, and drawing the tumor through the incision—which had to be slightly enlarged—revealed that the obstruction was due to volvulus at the sigmoid flexure. The gut was twisted upon itself and lapped over like the end of a tobacco twist. Adhesions so firm had taken place that the lumen of the bowel was entirely destroyed and a solid tumor remained in its place. The intestines were fearfully congested, dark and purple, and at the tumor almost black. The question as to the propriety of stitching the colon to the edges of the incision and immediately opening the gut, or of resecting the tumor, closing up the rectal end, dropping it back into the cavity, and stitching the other cut end to the incision, at once presented itself. At this juncture, in manipulating the tumor and testing the firmness of the adhesions, a slight tear was made in the gut immediately above the tumor, through which a small quantity of liquid fecal matter escaped. This settled the question. The colon was immediately clamped above and below the tumor—phimosis forceps, covered with rubber tubing, being used for clamps—about two inches above and an inch and one-half below the tumor. The rectal end of the intestine was cut through, its edges were turned in and stitched over with Lembert sutures of fine silk. This was dusted over with iodoform and dropped back into the cavity. The same procedure was gone through with above the tumor, entirely removing it. The cut edges of the colon were carefully stitched to the walls of the incision. Upon removal of the clamp, there was a gush of liquid fecal matter through the opening, which continued for some little time. The abdominal cavity was irrigated with hot water through the opening left for the removal of the clamp and sponge, until the water returned clear and pure. The opening was then closed, and a pad of iodoform gauze was placed over the artificial anus, borated cotton covering this, kept in place by a many-tailed binder, the use of which was suggested by my friend, Dr. L. S. McMurtry. The patient was then put to bed with hot bottles placed around him at intervals. Reaction was fairly good, and the next morning the patient expressed his thanks for the relief afforded him, having had no pain since the operation. His condition, however, was not promising. Pulse, 140; temperature, 100; respiration, 36. Abdominal tenderness was not marked, but there seemed to be a condition of exhaustion which, instead of growing better, gradually increased until death took place at 9:50 P. M., June 20th, thirty-one hours after the operation.

In regard to the treatment of this case before I saw it, comment is unnecessary. The question is, whether, under the circumstances, the operation was a justifiable one, and whether the resection of gut, with the closing of the rectal end and dropping it into the cavity, and the formation of an artificial anus at the incision, after the manner of Madelung, was to be preferred to stitching the colon to the walls of the incision, and making an immediate opening into the gut, without regard to the tumor formed by inflammatory adhesions. Taking everything into consideration, the almost gangrenous condition of the bowel at the site of the obstruction; the tear made, through which fecal matter had escaped; the doubt which existed that there was sufficient integrity in the wall of the gut to insure the holding of the stitches; fear of leaving a gangrenous tumor within the cavity; all of which were potent factors in favor of the graver operation, I feel justified in the course pursued, even if nothing more was accomplished than the relief of the patient from the horrible agony which he was suffering.

Some general remarks on the operation of colotomy may not be out of place here, especially as there seems to be a difference of opinion among surgeons in regard to the preferability of the two methods—lumbar and inguinal.

The subject of colotomy, always one of interest, has, during the past decade, demanded much attention from the surgical world. The revival of Littre's operation, so long debarred by that bugbear of abdominal surgery, the dread of opening the peritoneum, has made it necessary to compare the merits of the extra, so-called, and intra-peritoneal procedures. Littre, in 1710, first proposed to open the sigmoid flexure for the relief of imperforate anus in children, but as to his ever having actually performed the operation admits of a doubt ; at all events, it seems to have been forgotten till revived by Pillore, of Rouen, in 1776, who proceeded by a different method, opening the cæcum by an incision made in the right inguinal region. In 1796, Callisen suggested an operation whereby the colon might be entered without opening the peritoneal cavity. He experimented on the cadaver, with a view of exposing the bowel where it was not covered by the peritoneum, by a vertical incision in the left lumbar region ; failing in this, he made an attempt to carry out his idea on the living. To Amussat unquestionably belongs the credit of having first actually performed the retro-peritoneal operation, which was done upon the right side by a transverse incision, with the result of five successes in six cases. In this country, Ashmead, of Philadelphia, did a left lumbar retro-peritoneal colotomy in 1842, being unaware at the time of his operation of Callisen's proposal.[1] Until recently, the operation, as usually performed, has been a combination of the methods of Callisen and Amussat. Like Callisen's, it was done on the left side ; and, like Amussat's, it was carried out through an incision which is either transverse, or obliquely transverse. The oblique incision, first recommended by Bryant, is that now adopted by most surgeons. Colotomy may be performed for any condition which obstructs the passage of feces along the colon, or under any circumstances in which it is advisable to set that bowel at rest. Obstruction may be produced by various causes; such as cancer of the rectum or sigmoid flexure, or any other part of the colon ; tumors of the peritoneum, or any abdominal organ pressing on the bowel ; volvulus of the sigmoid flexure, or of the cæcum or ascending colon; fecal accumulations and collections of foreign matter which cannot be disturbed by other means. It may be called for in cases of incurable ulceration of the bowel, however induced, when we have reason to believe that irritation of feces and unrest of the intestinal walls contribute to the continuance of the disease; and in cases of extreme dilatation, with atony of the colon, giving rise to frequent attacks of obstruction.

As a measure intended to ward off imminent death, colotomy is called for in all cases of obstruction in the colon from whatever cause arising. For imperforate anus, the operation holds a special position. It is intended to prevent impending death, but it may or may not be regarded as a cure for the disease. In many cases it is the first step in the process of cure. In every infant born with imperforate anus, an operation of a local nature is first attempted; if this fails, colotomy by some method is performed, to ward off death; later on, an attempt may be made to get the bowel to discharge through the anus. In few words, it may be said that the indications to operate in any given case depend, in the first place, on the chance which the patient has of getting well without operation; and in the second place, upon the degree of probability with which success will follow the operation. To cases of acute obstruction of the sigmoid flexure, or elsewhere, there is practically but one termination—death. No case of volvulus, whether of large or small intestine, has, as yet, been known to recover under treatment purely medicinal. Here, then, the indication is clear enough, as clear, Greig Smith[2] says, as the in dications to tie a bleeding carotid-operation. But it may be said that in the case reported, while the patient had no chance of getting well without operation, yet there was no probability that success would follow the operation, owing to the lapse of time since the attack began. Of course, it was no fault of mine that the operation was not done sooner, and the statement was made that there was very little, if any, chance of saving life, but that the relief from the intense pain was certain, and under such conditions the operation was proposed and consented to. That it accomplished all that I expected is unquestionably true, for from the moment that the patient came from under the influence of the anæsthetic until death took place there was no pain.

Now, as to the comparative merits of the two operations: As I have said before, the operation of inguinal colotomy is not new.

One hundred and eighty years ago it was suggested, but it has been left to modern surgery to simplify the procedure, to lay down fixed rules for its performance, and to carry it almost certainly to a successful issue. The indications for its performance are the same as those calling for the lumbar method, which, in point of origin, it preceded, and which I am almost certain it will supersede. In a recent paper[3] I find that so good a man as our President, in speaking of colotomy, uses the following language : "It is not the purpose of this paper to discuss the question *pro* and *con*, but it is safe to say, *that with English and American surgeons only one operation—lumbar colotomy—is looked upon as justifiable.* (Italics my own.)

In view of the fact that such surgeons as the Allinghams, Sr. and Jr., Treves, Chavasse, Reeves, Kelsey, Cripps and others advocate the inguinal in preference to the lumbar operation, this statement must be looked upon as a little sweeping, to say the least of it. Dr. Mathews further says, "It does not seem at all plausible to say that, with any method of operating, as much safety can be had in opening the peritoneum as in not opening it. If antiseptic surgery makes inguinal colotomy so very safe, why is it not logic to say that it also renders lumbar operation doubly safe as compared with other ways." Is it true that in the lumbar operation the peritoneum is never opened ? On the contrary, is it not true that in the large majority of the cases the peritoneum *is opened*? H. W. Allingham, Jr., in an interesting paper on the causes of failure to find the colon in the operation of lumbar colotomy and the way to obviate them,[4] says : The difficulties sometimes met in finding the large bowel and the occasional cases in which serious errors have been made are known to all ; and all will agree that unless one of the longitudinal muscular bands, which are invariably and only found in the large intestine, be seen, the intestine should not be opened from the loin. These bands are described as being situated, one on the anterior surface, another along the inner part, and the third at the posterior aspect of the gut. It is this posterior band that is looked for and generally supposed to be seen when searching for the bowel in the lumbar region. It is thought by some authorities that these bands can be easily detected without opening the peritoneum, but this is not so, except in a very few cases. The author finds from examination and dissection of over one hundred ascending and descending colons that the bands are always more easily and distinctly seen when they are covered by the peritoneum, which makes them hard, prominent, and shiny; whereas when the peritoneum is stripped off them these characteristics are lost. He admits that in eight cases out of one hundred examined, one or two of these bands could be seen, but not very distinctly, on the posterior part of the intestine, although they were uncovered by the peritoneum. When the peritoneum only covers about one-half or two-thirds of the circumference of the gut, it is generally reflected off the gut at the longitudinal bands on to the walls of the belly. Thus, unless the peritoneum is stripped off, the bands are not visible. If an attempt is made to expose the longitudinal fibers, the peritoneum, owing to its being so firmly adherent to them, is frequently torn and the peritoneal cavity opened, perhaps unknown to the operator. It is argued, in favor of lumbar colotomy, that the large intestine can be searched without opening the abdominal cavity. This, of course, is possible, yet it is much more important to make certain that the large intestine is being opened by first seeing the longitudinal bands. *This from the anatomical points mentioned, can only be done by opening the peritoneum.* (Italics my own.) However, Mr. Allingham proposes to prove that in this way only can the large intestine be found with certainty in most cases. He is strengthened in these conclusions by three cases in which he operated on the right side in the dead subject, where it afterwards appeared that if he had not looked carefully for the longitudinal bands the descending portion of the duodeum would have been opened instead of the large intestine. This occasionally happens in operating on the living. Again the position of the colon may be an abnormal one. Treves found the colon to be out of its uaual position in twenty-six cases out of a hundred on the right side, and in thirty-six cases out of a hundred on the left side. Allingham finds it to be the case in eleven cases out of sixty on the right side, and ten out of sixty on the left side. The percentage is eighteen and one-third cases out of one hundred on the right side, and sixteen and two-thirds out of one hundred on the left side. From this it would appear that the normal position of the gut is less common than generally supposed. With the intestine in its general position and a longitudinal band can be seen, uncovered by peritoneum, all should go well in the lumbar operation. But when no bands can be seen, the best distinction between large and small intestine is wanting, and Mr. Allingham considers it much more advisable to open the peritoneum intentionally and search for a piece of intestine with longitudinal bands, than to run the risk of opening the small intestine under the impression that the peritoneum has never been opened at all, and that it is the large intestine which is being dealt with. Mr. Allingham further says: "The colon may be entirely surrounded by firmly adherent peritoneum, and with a comparatively short mesentery, in which condition it is absolutely impossible to reach it or to see the ongitudinal bands without first opening the peritoneal cavity." The ascending and descending colons were found by Mr. Treves to have a varying length in twenty-six cases out of one hundred on the right, and in thirty-six cases out of one hundred on the left side. Mr. Allingham observed this in forty-nine cases out of sixty on the right side, and in fifty out of sixty on the left. The percentage is eighty and two-thirds cases out of one hundred on the right, and eighty-three and one-third out of one hundred on the left. In cases where there is great length of mesentery, the intestine, though it may rest in the loin, can so alter its position in the belly that when operating on either side it may lie on the side of the belly opposite to that in which the incision is made. It then, in the cases reported, was said and supposed to be impossible to find the colon from the lumbar region. Such cases show how imperative it is to make sure that it is the large and not the small intestine, or even the stomach which is going to be opened. The presence of the appendices epiploicæ may inform the surgeon that he has found the large intestine, but these are not considered as important as the longitudinal bands, since they may not always exist on the piece brought to view. Mr. Allingham does not at all advocate lumbar colotomy, when it is possible to perform the inguinal operation, for the lumbar is certainly the more difficult, the patient runs greater risk, and recovers less quickly, and the after results are not as satisfactory. Kelsey says, "Allingham does not emphasize the point which he renders so plain, that when the lumbar operation is performed with these necessary precautions to make sure that the colon is the part opened, it loses its only supposed advantage over the inguinal—the non-interference with the peritoneal cavity." Mr. Allingham gives the following reasons why he thinks inguinal colotomy is preferable to lumbar colotomy :[5]

1st. The position of the patient is better at the time of operation for the patient, the operator and the anæsthetist.

2d. There is not so much tendency for the gut to fall away from the wound, either at the time or after the operation.

3d. The intestine is easier to find, especially so on account of the incision being made much higher than usual. The result of five hundred post mortem examinations is quoted to verify this statement.

4th. The feces do not pass below the artificial opening if a good spur be made.

5th. There is less constitutional disturbance.

6th. There is little or no suppuration.

7th. The tendency for the opening to contract is not greater. Chavasse, of Birmingham, advances the following reasons why he prefers the inguinal to the lumbar operation.[6]

1st. It is readily performed.

2d. The patient is readily able to attend to his or her wants in connection with false anus.

3d. The patient is able to lie on his back without discomfort.

4th. In malignant disease four or five inches more of the colon are left for it to perform its duties.

5th. Being nearer the seat of the disease, the operator is able to ascertain, if necessary, the precise limits of the growth. As a matter of practical experience, he states that he has always found that the opening in the sigmoid flexure has been sufficiently remote from the neoplasm as not to become implicated during life.

Malignant diseases of the flexure, except at its juncture with the rectum, is of rare occurrence. Up to this time he had performed inguinal colotomy thirteen times without a death. Cripps'[7] records the results obtained by him in thirty-seven operations, fifteen of which were lumbar and twenty-two inguinal, with two deaths, a mortality of rather more than five per cent. The mortality of the operation has been very great; the analysis of collected cases (244) of lumbar colotomy, made by Batt in 1884, gave a mortality of thirty-two per cent. The inguinal operation gave a mortality of over fifty per cent. These statistics, he thought, represented the results up to that period, but were misleading as affording any indication of what may be expected of the operation, at the present time, under favorable circumstances. He regards colotomy as an operation of great delicacy, requiring good anatomical knowledge with trained manipulative skill. The preparation of the patient, the hygienic surroundings and the subsequent treatment of the wound all demand most careful consideration, and materially influence the result. The chief objections to the lumbar operation were stated to be—

1st. The absence of sufficient working space between the lower border of the last rib and crest of the ilium.

2d. Difficulty in the identification of the bowel in the limited space; the longitudinal bands are sometimes impossible to recognize; numerous instances are recorded where the small bowel, the duodenum, or even the stomach have been opened by mistake.

3d. In fat or muscular patients, difficulty owing to the depth of the bowel and its want of mobility in fixing to the skin without undue tension.

4th. Abnormal deviations of the bowel, rendering it impossible to find it by this incision.

5th. Inconveniences of the opening behind for cleanliness and adjustment of pads.

Inguinal colotomy meets all these objections by affording a space in front, practically unlimited, through an incision in which the bowel can be carefully inspected and identified by its longitudinal bands, its convoluted surface, and its glandular epiploicæ. The mobility of the sigmoid flexure and laxity of the skin remove any difficulty in fixing the bowel without undue tension. The ease with which thorough exploration of the cavity can be made through the incision, removes all difficulties attending abnormal course of the colon. This method possesses also an advantage in enabling the surgeon to verify the diagnosis by free exploration. The objections urged against the methods are the tendency for prolapse of the bowel which occurs, and that it is unsuitable for urgent cases. The first can be overcome by drawing down the bowel to its full extent, and the danger in the second is believed to be more imaginary than real. Madelung[8] recommends that the colon be completely cut through; the lower opening being closed and returned, the upper opening being sutured to the skin. At the British Medical Association at Leeds, Mr. F. Marsh showed a patient on whom he had performed Madelung's operation

Fig. 1.—British Medical Journal.

with a very satisfactory result. Cripps'[9] operation is performed as follows: The patient having been carefully prepared by a bath and cleansing of the operative surface, an incision two and one-half inches is made at right angles across an imaginary line, drawn from the anterior superior spine to the umbilicus, and one and one-half inches from the superior spine. (Fig. 1.) In order to make the opening somewhat vulvular, the skin should be drawn a little inward and the tissues divided until the peritoneum is reached, when this should be picked up and incised to nearly the full length of the cutaneous incision. The colon being found, a loop of it is drawn into the wound, and, if loose folds of the sigmoid flexure remain immediately above the opening, it should be drawn down and passed through the fingers into the cavity, at the lower angle. When all has passed, two provisional ligatures of stout silk are passed through the longitudinal muscular bands opposite the mesenteric attachment, two inches apart. The bowel is now temporarily returned to the cavity, and the parietal peritoneum is sutured to the skin on each side of the incision by two sutures of fine Chinese silk one and a half inches

Fig. 2.—British Medical Journal.

Fig. 3.—British Medical Journal.

apart (Fig. 2), after which the bowel is fixed to the skin and parietal peritoneum by seven or eight fine sutures in each side, the last at each angle going across from one side to the other, and should be so attached as to have two-thirds of its circumference external to the sutures. The sutures for the lower side should be passed through the lower longitudinal band, as it is a strong portion of the bowel. Those for the upper should be inserted close to the mesenteric attachment. (Fig. 3.) It is best to pass all of the sutures and then tie them in order, with moderate tension. In urgent cases, the bowel can be opened at once; if not, the opening may be delayed until the fifth or sixth day, when it is usually found to be covered with a layer of lymph of surprising thickness.

The provisional ligatures will be found a useful guide, the bowel being opened to the full length between them, and the superfluous flaps on either side trimmed off with scissors to the level of the skin. All sutures may be removed by the ninth day, or earlier if there is redness around them. If the bowel is not opened immediately, a piece of protective should be placed over it to prevent adhesions of the granulations to the gauze, the wound dressed antiseptically, with an additional thick pad and a broad flannel bandage firmly applied. This is important in order to prevent tearing out of the sutures in case vomiting should occur. Firm pressure by the nurse over the wound is of great value at this time.

REFERENCES.

(1) Greig Smith, abdominal surgery, 2d edition.
(2) Greig Smith, abdominal surgery, 2d edition.
(3) Medical Progress, July, 1890.
(4) Sajous Annual, Vol. III., 1889.
(5) Annals of Surgery, Vol. VII., p. 460.
(6) London Lancet, February, 1889.
(7) Sajous Annual, Vol. III., 1890.
(8) International Medical Annual, 1890.
(9) British Medical Journal, April, 1889.

## Figures and Tables

**Fig. 1. f1:**
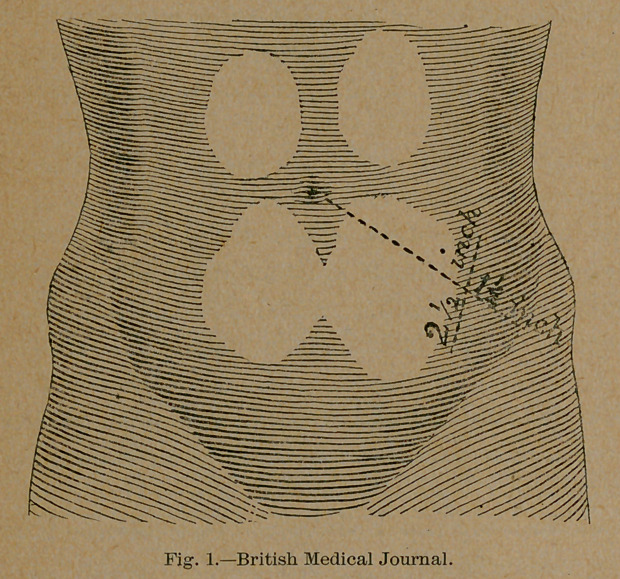


**Fig. 2. f2:**
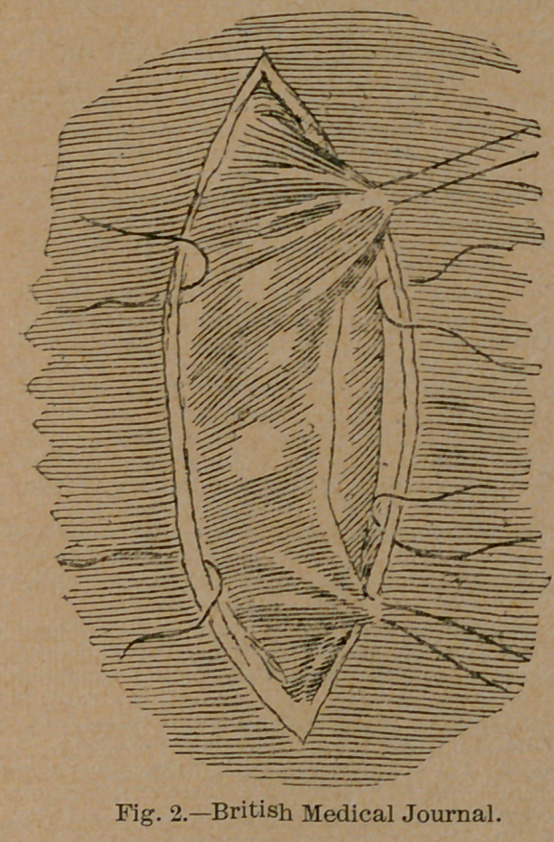


**Fig. 3. f3:**